# The Impact of Selenium Deficiency and Supplementation on Hashimoto’s Thyroiditis: A Clinical Evaluation

**DOI:** 10.2174/0118715303424962251204064911

**Published:** 2026-04-28

**Authors:** Hasan Acik, Yusuf Aydin

**Affiliations:** 1 Department of Internal Medicine, Faculty of Medicine, Okan University, Istanbul, Turkiye;; 2 Department of Endocrinology and Metabolic Disorders, Okan University, Istanbul, Turkiye

**Keywords:** Hashimoto thyroiditis, selenium, thyroid function, autoimmunity, supplementation, thyroid antibodies

## Abstract

**Introduction:**

Hashimoto thyroiditis (HT) is an autoimmune disorder that leads to hypothyroidism. Selenium (Se) is essential for thyroid hormone metabolism and immune function, but its role in HT remains debated. This study investigates Se levels in HT patients and evaluates the impact of supplementation on thyroid autoimmunity and thyroid function.

**Methods:**

This retrospective study analyzed HT patients and healthy controls. Biochemical parameters, including Se, thyroid-stimulating hormone (TSH), free triiodothyronine (FT3), free thyroxine (FT4), anti-thyroid peroxidase (anti-TPO), and anti-thyroglobulin (anti-Tg) antibodies, were compared. Se-deficient HT patients (<80 µg/L) received selenium supplementation for six months. Paired t-tests and Spearman correlation analyses were used for statistical evaluation.

**Results:**

Among 270 HT patients and 350 controls, TSH levels were significantly elevated in HT (*p*<0.001), whereas Se levels showed no significant baseline difference (*p*=0.87). In Se-deficient patients (n=60), supplementation increased Se levels from 68.60 ± 12.10 µg/L to 104.92 ± 34.45 µg/L (*p*<0.001). However, anti-TPO and anti-Tg levels increased after treatment (*p*=0.01 and *p*=0.03, respectively). No significant changes were observed in TSH (*p*=0.27), FT3 (*p*=0.20), or FT4 (*p*=0.19). Se positively correlated with anti-TPO (r=0.36, *p*=0.0102) and negatively with FT3 (r=-0.39, *p*=0.0046).

**Discussion:**

Selenium supplementation restored serum Se levels but was unexpectedly associated with increased antibody titers. This finding contrasts with several randomized trials reporting antibody reduction, suggesting that baseline Se status, the form of supplementation, or differences in study design may account for the discrepancy. The observed correlations should be interpreted with caution.

**Conclusion:**

Selenium supplementation normalizes selenium status in HT patients, but its effects on thyroid autoimmunity and thyroid function remain uncertain. Long-term randomized controlled trials are needed to determine its therapeutic value.

## 1 INTRODUCTION

Hashimoto's thyroiditis (HT) is the most prevalent cause of hypothyroidism in iodine-sufficient regions, affecting approximately 160 million individuals worldwide [1]. The disease is characterized by lymphocytic infiltration of the thyroid gland, leading to gradual destruction of the follicular cells and a progressive decline in thyroid function. HT predominantly affects females, with a female-to-male ratio ranging from 4:1 to 10:1, suggesting a potential role for hormonal and genetic factors in disease susceptibility. The pathophysiology of HT involves complex interactions between genetic predisposition, environmental triggers, and immune dysregulation [2, 3].

The autoimmune mechanism underlying HT is driven by the breakdown of immune tolerance to thyroid antigens.

The presence of thyroid peroxidase antibodies (TPOAb) and thyroglobulin antibodies (TgAbs) is a hallmark of this disease, indicating an ongoing immune-mediated attack on the thyroid gland. Cytotoxic T lymphocytes infiltrate the thyroid tissue, leading to the production of pro-inflammatory cytokines, oxidative stress, and apoptosis of thyrocytes. Over time, glandular atrophy and hypothyroidism can occur. Although the precise triggers of autoimmunity remain elusive, iodine intake, infections, gut microbiome alterations, and genetic factors have been implicated in the initiation and progression of the disease [4, 5].

Selenium (Se) is an essential trace element that plays a critical role in thyroid homeostasis. The thyroid gland contains the highest concentration of Se per gram of tissue, which is required for the synthesis of selenoproteins, including glutathione peroxidases (GPXs), thioredoxin reductases (TXNRDs), and iodothyronine deiodinases (DIOs) [6]. These enzymes are fundamental in mitigating oxidative stress, modulating immune responses, and regulating the metabolism of thyroid hormones. The antioxidant properties of Se-containing enzymes protect thyrocytes from hydrogen peroxide-mediated damage, a natural byproduct of thyroid hormone synthesis. Furthermore, deiodinases catalyze the conversion of thyroxine (T4) to its active form, triiodothyronine (T3), rendering Se indispensable for thyroid hormone production [6, 7].

The potential association between Se and thyroid autoimmunity has garnered increasing attention in recent years. Epidemiological studies have reported an inverse relationship between Se levels and HT prevalence. Se deficiency is associated with increased TPOAb titers and a higher risk of HT development. Mechanistically, inadequate Se levels may impair antioxidant defenses, leading to excessive production of reactive oxygen species (ROS), which exacerbates inflammatory pathways and antigen production. Moreover, Se influences immune function by modulating T-helper (Th) cell differentiation, particularly the balance between Th1 and Th2 responses, which plays a crucial role in HT pathogenesis [8, 9].

Given the potential role of Se in thyroid autoimmunity, several randomized controlled trials (RCTs) have evaluated the effects of Se supplementation in patients with HT. While some studies have reported significant reductions in TPOAb levels and improved QoL, others have found no substantial benefits. These discrepancies may be attributed to differences in baseline Se status, supplementation dose, treatment duration, and study populations. Despite conflicting evidence, meta-analyses have suggested that Se supplementation may provide benefits in Se-deficient individuals, particularly by reducing thyroid autoantibody levels and oxidative stress markers [10, 11].

Recent large-scale reviews have provided updated insights into the role of Se supplementation in autoimmune thyroiditis. A systematic review and meta-analysis of randomized clinical trials demonstrated heterogeneous outcomes, with antibody reduction primarily observed in selenium-deficient subgroups [12]. Similarly, another meta-analysis emphasized that baseline selenium status substantially influences treatment response [13], whereas a network meta-analysis comparing different supplementation strategies highlighted the need for standardized dosing [14]. Collectively, these findings reinforce the notion that the effects of selenium in Hashimoto’s thyroiditis are context-dependent and may not be universally beneficial.

Despite these emerging data, regional evidence from Turkish cohorts is scarce. Given the variability in baseline nutritional status, dietary iodine intake, and treatment practices across populations, further research is needed to clarify the clinical significance of selenium supplementation in Hashimoto’s thyroiditis. Therefore, this study was designed to evaluate selenium levels in patients with HT, compare them with those of healthy individuals, and assess the impact of supplementation on thyroid autoimmunity and thyroid function in a Turkish population.

## 2 MATERIALS AND METHODS

### 2.1 Study Design and Type of Study

A retrospective observational analysis was performed at the Internal Medicine Clinic of a university hospital. Patient records of individuals diagnosed with HT and monitored at our facility were retrospectively examined. Prior to commencing the study, ethical clearance was obtained from the ethics committee. Among the 86 patients identified with selenium deficiency, only 60 were included in the supplementation analysis due to completion of follow-up and availability of paired laboratory data.

### 2.2 Study Population

Patients diagnosed with HT based on clinical, laboratory, and ultrasonographic findings were included. Data were collected from electronic medical records between January 2018 and December 2023. As this was a retrospective observational study, the sample size was determined based on the number of eligible patients with complete records during the study period. No formal power calculation was performed, consistent with the methodological standards for retrospective analyses.

### 2.3 Inclusion and Exclusion Criteria

The study included individuals aged ≥ 18 years with a confirmed diagnosis of HT. Participants were required to have comprehensive medical records detailing their baseline and post-selenium supplementation laboratory results. The selection process focused on patients who received standard HT without additional immunomodulatory interventions. The study excluded patients with concurrent autoimmune conditions, such as Graves' disease, systemic lupus erythematosus, or rheumatoid arthritis. Individuals with a history of thyroidectomy, radioactive iodine therapy, or chronic kidney or liver disease affecting selenium metabolism were also excluded. Women who were pregnant or breastfeeding were also excluded.

### 2.4 Data Collection and Parameters

Demographic, biochemical, and clinical parameters were extracted from medical records. The demographic variables included age, sex, and body mass index (BMI). Thyroid function and autoimmune markers assessed included thyroid-stimulating hormone (TSH), free T3 (FT3), free T4 (FT4), thyroid peroxidase antibodies (anti-TPO), thyroglobulin antibodies (anti-Tg), and selenium levels.

### 2.5 Intervention

Patients with selenium deficiency (defined as serum selenium <80 μg/L) received selenium supplementation (20 μg/day of sodium selenite in drop form or 200 μg/day of selenomethionine in oral form) for six months. Post-treatment thyroid function and antibody levels were reassessed. Among these patients, 40 received 20 μg/day sodium selenite drops and 20 received 200 μg/day selenomethionine (SeMet) orally, based on clinician preference. Subgroup comparisons did not reveal significant differences; however, the limited sample size reduced statistical power.

### 2.6 Statistical Analysis

Continuous variables are presented as mean ± standard deviation (SD) or median (interquartile range), depending on the distribution assessed using the Shapiro–Wilk test. Categorical variables were analyzed using the chi-square (χ^2^) test. Paired comparisons of pre- and post-supplementation data were performed using the Wilcoxon signed-rank test or the paired t-test. Spearman’s correlation was used to assess the relationship between selenium levels and thyroid markers. For categorical variables, chi-square (χ^2^) values and corresponding *p*-values were reported. Additionally, a post-hoc power analysis indicated that with 60 participants, the study had approximately 72% power to detect a medium effect size (Cohen’s d = 0.5) at α = 0.05. Statistical significance was set at *p* < 0.05.

## 3 RESULTS

A total of 270 patients with HT were included in this study. For comparative analysis, a healthy control group of 350 age-matched individuals was selected. The proportion of females was significantly higher in the HT group (83.8% *vs*. 77%, *p*=0.03); however, no significant differences were observed in selenium levels (*p*=0.87) between the groups or in the proportion of individuals with low selenium levels (*p*=0.96). TSH levels were significantly higher in the HT group (*p*<0.001), whereas free T3 and T4 levels did not differ significantly between the groups (*p*=0.53 and *p*=0.94, respectively). Comparisons of demographic, biochemical, and thyroid function parameters between healthy individuals and patients with HT are presented in Table **1**.

Among patients with HT and selenium deficiency, 86 were identified, of whom 60 received selenium replacement therapy and had complete post-treatment follow-up data.

Following selenium supplementation, serum selenium levels significantly increased from 68.60 ± 12.10 µg/L to 104.92 ± 34.45 µg/L (*p*<0.001). Anti-TPO levels exhibited a statistically significant increase from 190.39 ± 193.15 U/mL to 215.95 ± 212.18 U/mL (*p*=0.01), and anti-Tg levels also increased significantly from 415.19 ± 941.74 U/mL to 501.40 ± 1192.24 U/mL (*p*=0.03). A comparison of the pre- and post-treatment values is summarized in Table **2**.

Correlation analysis was performed to evaluate the relationship between selenium levels and thyroid function. A significant positive correlation was observed between selenium and anti-TPO levels (r = 0.36, *p* = 0.0102), whereas a significant negative correlation was observed with free T3 (r = –0.39, *p* = 0.0046). No significant correlations were found between selenium and TSH (r = –0.15, *p* = 0.2894) or free thyroxine (T4) (r = 0.05, *p* = 0.7438). The correlation between serum selenium levels and thyroid function parameters, including TSH, Anti-TPO, Free T3, and Free T4, in patients with HT is illustrated in Fig. (**1**).

## 4 DISCUSSION

This study explored the association between selenium levels and thyroid function in individuals with Hashimoto’s thyroiditis (HT) and examined the influence of selenium supplementation on thyroid autoimmunity. These findings highlight the complex relationship between selenium homeostasis, thyroid hormone metabolism, and autoimmune processes, contributing to the ongoing discussion on the potential clinical benefits of selenium supplementation in HT management.

Epidemiological research underscores the essential role of selenium in thyroid physiology, particularly due to its involvement in selenoprotein synthesis, which regulates oxidative stress and immune function [15, 16]. This study did not reveal a statistically significant difference in selenium levels between patients with HT and healthy controls, aligning with prior reports indicating that selenium deficiency is not universally observed in HT. However, selenium supplementation effectively increased serum selenium concentrations, confirming its role in restoring selenium status in deficient individuals. The unexpected increase in antibody titers after supplementation may be explained by the biphasic immunological actions of selenium. While selenium reduces oxidative stress *via* selenoproteins, excessive or imbalanced supplementation may transiently activate immune pathways, resulting in increased antibody production. Potential analytical variability in antibody assays, as well as unmeasured confounders such as iodine intake or concurrent medications, may have also contributed to these findings. These mechanisms underscore the complexity of selenium’s role in thyroid autoimmunity, as emphasized in recent reviews [12, 17].

One notable observation was the significant elevation in anti-TPO and anti-Tg antibody levels after selenium supplementation. This aligns with studies suggesting that selenium may alter immune function, possibly leading to transient fluctuations in autoantibody titers [11, 18]. However, these results contrast with those of Wang *et al*. and Wichman *et al*., who reported a decline in thyroid autoantibodies with selenium supplementation [10, 19]. Variations in baseline selenium status, study design, and patient population may account for these discrepancies.

The impact of selenium supplementation on thyroid function remains debatable. In this study, TSH levels exhibited a downward trend after supplementation, but the change was not statistically significant. Similar results have been reported by Wang *et al*. and Mikulska *et al*., indicating that selenium may have only a minor influence on thyroid function [10, 20, 21]. Furthermore, the absence of significant changes in free T3 and T4 levels post-supplementation supports previous research suggesting that selenium does not directly modulate thyroid hormone synthesis [22].

Correlation analysis provides further insights into the role of selenium in thyroid function. A significant inverse relationship was observed between selenium and free T3 levels, suggesting a possible reduction in T4-to-T3 conversion with increasing selenium levels. This is consistent with the hypothesis that selenium modulates deiodinase activity, as proposed by Wichman *et al*. [19]. Additionally, the positive correlation between selenium and anti-TPO levels suggests that selenium may be involved in immune regulation, a finding supported by studies by Wang *et al*. and Hu *et al*. [10, 23]. However, the absence of a correlation between selenium and TSH or free T4 levels indicates that the regulatory effects of selenium on the thyroid are likely indirect. Although the negative correlation between selenium and free T3 was statistically significant, the effect size was modest and should be interpreted as exploratory rather than clinically definitive. Similar observations have been reported by Wang *et al*., who noted heterogeneity in thyroid hormone responses, and by Wichman *et al*., whose meta-analysis concluded that the evidence remains insufficient for consistent thyroid hormone modulation [10, 19]. Thus, the observed Se–FT3 association in our study must be regarded as hypothesis-generating.

While selenium supplementation remains an area of interest in HT therapy, its effects appear to be modulated by baseline selenium status and interindividual differences. Recent meta-analyses have further clarified the heterogeneity of outcomes associated with selenium supplementation in HT. Huwiler *et al*. demonstrated that antibody reduction was mainly observed in selenium-deficient subgroups, whereas Kong *et al*. highlighted baseline selenium status as a critical determinant of treatment response [12, 13]. Peng *et al*. used a network meta-analysis to compare supplementation strategies, emphasizing the variability across selenium forms and doses [14]. Collectively, these findings confirm that the clinical effects of selenium are context-dependent and cannot be generalized to all patients with HT. Future research should incorporate patient stratification based on baseline selenium levels to identify subgroups that may derive the greatest benefit from supplementation.

## 5 STUDY LIMITATIONS

This study has several limitations that must be acknowledged. First, the retrospective observational design inherently introduces selection bias and precludes the establishment of causal relationships. Among patients with selenium deficiency, only those with complete follow-up data were included, which may have further contributed to selection bias. Second, selenium supplementation was administered in two different chemical forms (sodium selenite and selenomethionine); however, the subgroup sizes were relatively small and underpowered to detect potential differences in clinical response. The supplementation group comprised only 60 patients, and although a post-hoc power analysis indicated approximately 72% power to detect a medium effect size, the study remained limited in its ability to assess smaller but clinically relevant changes in the outcome measures.

Potential unmeasured confounders, such as dietary iodine intake, food selenium content, concurrent medications, and gut microbiome composition, were not systematically evaluated and may have influenced antibody responses. The study follow-up period of six months was relatively short, and the long-term effects of supplementation on thyroid function and clinical outcomes could not be assessed. Finally, the unexpected increase in antibody titers after selenium supplementation highlights the complexity of immune modulation in Hashimoto’s thyroiditis and suggests that further large-scale prospective randomized controlled trials are required to clarify the long-term efficacy and safety of selenium supplementation in different patient subgroups.

## CONCLUSION

This study demonstrates that selenium supplementation effectively restores serum selenium levels in patients with Hashimoto’s thyroiditis who are selenium-deficient. However, supplementation was unexpectedly associated with increased anti-TPO and anti-Tg antibody levels, and no significant improvements were observed in thyroid hormone parameters. Correlation analysis revealed modest associations between selenium, free T3, and antibody titers, which should be considered exploratory, given the limited sample size. Taken together, these findings highlight the complex and context-dependent roles of selenium in thyroid autoimmunity. Long-term, adequately powered randomized controlled trials are required to clarify its clinical efficacy, optimal dosing strategies, and safety in different patient subgroups.

## Figures and Tables

**Fig. (1):**
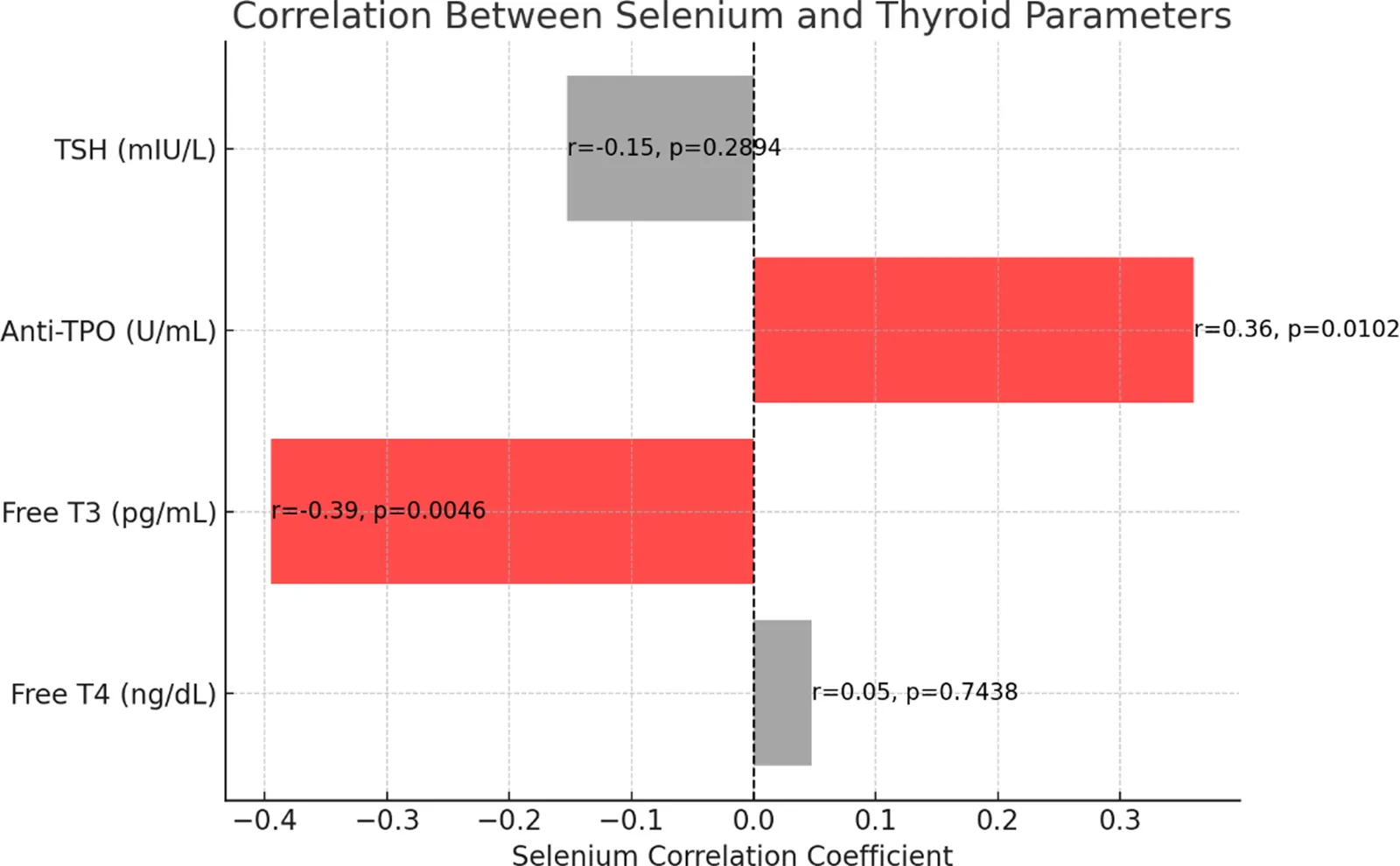
Correlation between selenium levels and thyroid parameters (TSH, anti-TPO, free T3, free T4) in patients with Hashimoto’s thyroiditis. Bars represent Spearman’s correlation coefficients (r) with corresponding *p*-values. Positive correlations are shown in red and negative correlations are shown in grey. The X-axis indicates the correlation coefficients, and the Y-axis lists the evaluated thyroid parameters. **Abbreviations:** TSH, Thyroid-Stimulating Hormone; Anti-TPO, Anti-Thyroid Peroxidase Antibody; FT3, Free Triiodothyronine; FT4, Free Thyroxine.

**Table 1:** Comparison of demographic, biochemical, and thyroid function parameters between healthy individuals and hashimoto’s thyroiditis patients.

**Parameter**	**Metric**	**Healthy Individuals (n=350)**	**Hashimoto’s Thyroiditis (HT) (n=270)**	**χ^2^ / t value**	***p*-value**
**Age (years)**	Mean ± SD	38.65 ± 10.03	39.59 ± 9.87	t = -1.15	0.25
**Gender**	Female, n (%)	270 (77.0)	227 (83.8)	χ^2^ = 4.68	0.03*
-	Male, n (%)	80 (23.0)	43 (16.2)	-	-
**Body Mass Index (kg/m^2^)**	Mean ± SD	23.29 ± 2.75	23.42 ± 2.70	t = -0.47	0.64
**Selenium (µg/L)**	Mean ± SD	77.03 ± 19.42	78.23 ± 23.35	t = -0.16	0.87
**Selenium Status**	Normal, n (%)	219 (62.6)	143 (62.4)	χ^2^ = 0.00	0.96
-	Low, n (%)	131 (37.4)	86 (37.6)	-	-
**Anti-TPO**	Negative, n (%)	326 (93.2)	0 (0)	χ^2^ = 531.2	*p*<0.001*
-	Positive, n (%)	24 (6.8)	229 (100)	-	-
**TSH (mIU/L)**	Mean ± SD	1.76 ± 1.78	3.95 ± 13.92	t = -3.84	*p*<0.001*
**Free T3 (pg/mL)**	Mean ± SD	2.82 ± 0.59	2.79 ± 0.64	t = 0.63	0.53
**Free T4 (ng/dL)**	Mean ± SD	0.98 ± 0.13	0.98 ± 0.18	t = -0.08	0.94

**Note:** Data are presented as mean ± standard deviation (SD) for continuous variables and as n (%) for categorical variables. An independent samples t-test was used for continuous variables, and the chi-square (χ^2^) test was applied for categorical comparisons.

**Abbreviations:** BMI, Body Mass Index; Anti-TPO, Anti-Thyroid Peroxidase Antibody; TSH, Thyroid-Stimulating Hormone; FT3, Free Triiodothyronine; FT4, Free Thyroxine.

**Table 2:** Pre- and post-replacement comparison of selenium levels, thyroid function, and autoimmune markers in hashimoto’s thyroiditis patients.

**Parameter**	**Pre-Treatment**	**Post-Treatment**	***p*-value**
**Selenium (µg/L)**	68.60 ± 12.10	104.92 ± 34.45	*p*<0.001
**Anti-TPO (U/mL)**	190.39 ± 193.15	215.95 ± 212.18	0.01
**Anti-Tg (U/mL)**	415.19 ± 941.74 Median (IQR): 185 (90–460)	501.40 ± 1192.24 Median (IQR): 210 (95–520)	0.03
**TSH (mIU/L)**	3.02 ± 4.63	2.22 ± 1.71	0.27
**Free T3 (pg/mL)**	2.76 ± 0.55	2.68 ± 0.47	0.20
**Free T4 (ng/dL)**	0.94 ± 0.12	0.98 ± 0.13	0.19

**Note:** Data are presented as mean ± standard deviation (SD) and median (interquartile range, IQR) where appropriate, depending on distribution (assessed by the Shapiro–Wilk test). Paired t-test was used for normally distributed variables, and the Wilcoxon signed-rank test for non-normally distributed variables.

**Abbreviations:** Anti-TPO, Anti-Thyroid Peroxidase Antibody; Anti-Tg, Anti-Thyroglobulin Antibody; TSH, Thyroid-Stimulating Hormone; FT3, Free Triiodothyronine; FT4, Free Thyroxine.

## Data Availability

The datasets generated and/or analyzed during the current study are available from the corresponding author on reasonable request.

## LIST OF ABBREVIATIONS

HT: Hashimoto thyroiditis
Se: Selenium
FT3: Free Triiodothyronine

## STANDARDS OF REPORTING

Strobe guidelines were followed.
